# Mango (*Mangifera indica* L.) Leaves: Nutritional Composition, Phytochemical Profile, and Health-Promoting Bioactivities

**DOI:** 10.3390/antiox10020299

**Published:** 2021-02-16

**Authors:** Manoj Kumar, Vivek Saurabh, Maharishi Tomar, Muzaffar Hasan, Sushil Changan, Minnu Sasi, Chirag Maheshwari, Uma Prajapati, Surinder Singh, Rakesh Kumar Prajapat, Sangram Dhumal, Sneh Punia, Ryszard Amarowicz, Mohamed Mekhemar

**Affiliations:** 1 Chemical and Biochemical Processing Division, ICAR—Central Institute for Research on Cotton Technology, Mumbai 400019, India; 2 Division of Food Science and Postharvest Technology, ICAR—Indian Agricultural Research Institute, New Delhi 110012, India; vivek_11593@iari.res.in (V.S.); uma_11103@iari.res.in (U.P.); 3 ICAR—Indian Grassland and Fodder Research Institute, Jhansi 284003, India; maharishi.tomar@icar.gov.in; 4 Agro Produce Processing Division, ICAR—Central Institute of Agricultural Engineering, Bhopal 462038, India; muzaffar.hasan@icar.gov.in; 5 Division of Crop Physiology, Biochemistry and Post-Harvest Technology, ICAR-Central Potato Research Institute, Shimla 171001, India; sushil.changan@icar.gov.in; 6 Division of Biochemistry, ICAR—Indian Agricultural Research Institute, New Delhi 110012, India; minnusasi1991@gmail.com; 7 Department of Agriculture Energy and Power, ICAR—Central Institute of Agricultural Engineering, Bhopal 462038, India; chirag.maheshwari@icar.gov.in; 8 Dr. S.S. Bhatnagar University Institute of Chemical Engineering and Technology, Panjab University, Chandigarh 160014, India; ssbhinder@pu.ac.in; 9 School of Agriculture, Suresh Gyan Vihar University, Jaipur 302017, Rajasthan, India; rakesh.prajapat@mygyanvihar.com; 10 Division of Horticulture, RCSM College of Agriculture, Kolhapur 416004, Maharashtra, India; sdhumal@msu.edu; 11 Department of Food, Nutrition, & packaging Sciences, Clemson University, Clemson, SC 29634, USA; snehpunia69@gmail.com; 12 Institute of Animal Reproduction and Food Research, Polish Academy of Sciences, 10-748 Olsztyn, Tuwima 10, Poland; amaro@pan.olsztyn.pl; 13 Clinic for Conservative Dentistry and Periodontology, School of Dental Medicine, Christian-Albrecht’s University, 24105 Kiel, Germany

**Keywords:** mango leaves, biological activities, phenolic bioactives, polysaccharides, health promoting effects

## Abstract

*Mangifera indica* L. belongs to the family of Anacardiaceae and is an important fruit from South and Southeast Asia. India, China, Thailand, Indonesia, Pakistan, Mexico, Brazil, Bangladesh, Nigeria, and the Philippines are among the top mango producer countries. Leaves of the mango plant have been studied for their health benefits, which are attributed to a plethora of phytochemicals such as mangiferin, followed by phenolic acids, benzophenones, and other antioxidants such as flavonoids, ascorbic acid, carotenoids, and tocopherols. The extracts from mango leaves (MLs) have been studied for their biological activities, including anti-cancer, anti-diabetic, anti-oxidant, anti-microbial, anti-obesity, lipid-lowering, hepato-protection, and anti-diarrheal. In the present review, we have elaborated on the nutritional and phytochemical profile of the MLs. Further, various bioactivities of the ML extracts are also critically discussed. Considering the phytochemical profile and beneficial effects of the MLs, they can be used as a potential ingredient for the development of functional foods and pharmaceutical drugs. However, more detailed clinical trials still needed to be conducted for establishing the actual efficacy of the ML extracts.

## 1. Introduction

Mango (*Mangifera indica* L.) ascribed to the family Anacardiaceae has been adjudged as the vital traditionally significant and one of the most economically important tropical fruit crop globally [1]. Mango is an evergreen tree with a lot of traditional medicinal resources apart from its very famous fruits. Mangoes are native to the South and Southeast Asia, and in 2018, the global production of mangoes (the report includes guavas and mangosteens) was 55.4 million tonnes. The largest mango producing countries are India, China, Thailand, Indonesia, Pakistan, Mexico, Brazil, Bangladesh, Nigeria, and the Philippines. Apart from its economically important portion (fruit), large amounts of crop residues such as leaves, flowers, stem, and bark are generated during pruning, which causes complications of disposal to the farmers. Mango leaves (MLs) are the potential source of minerals, viz. nitrogen, potassium, phosphorus, iron, sodium, calcium, magnesium, and vitamins, viz. A, B, E, and C. A major bio-macromolecule present in mango leaves is protein. MLs can be utilized as an alternative source of livestock feeding in developing countries for alleviating the food shortage for livestock.

Extracts of the MLs have been utilized for traditional medicines to cure diabetes, bronchitis, diarrhea, asthma, kidney, scabies, respiratory problems, syphilis, and urinary disorders [2,3]. The most active biological constituent of MLs is mangiferin, followed by phenolic acids, benzophenones, and other antioxidants such as flavonoids, carotenoids, quercetin, isoquercetin, ascorbic acid, and tocopherols. Mangiferin is the main contributor of most of the biological activities of MLs extract. MLs have a great scope of valorization as they are recognized to possess varied phytochemical, biological, and pharmacological properties, viz. anti-microbial, antioxidant, anti-diabetic, anti-tumour, and immunomodulatory effects. ML oil (MLO) contains monoterpenes, sesquiterpenes, minor quantities of other analogues, and trace amounts of non-terpenoid hydrocarbons and oxygenated hydrocarbons. The essential oil from MLs also possesses bacteriostatic properties and contains several antimicrobial constituents such as α-gurjunene, trans-caryophyllene, α-humulene, α-selinene, and camphor. The benzophenone derivatives in MLs possess significant *α*-glucosidase inhibitory and immunosuppressive activities. There are several reviews that have been developed to discuss the bioactive compounds and health promoting effect of mango fruit/pulp [4,5,6,7], whereas others contain a scattered compilation of literature on mango seeds, MLs, and mango bark [8,9].

There is no critical compilation on the crucial information on MLs’ bioactives and associated bioactivities. Therefore, the current review will be focused on the nutritional and phytochemical profile of the MLs. The review also delivers important health promoting activities of the MLs extracts.

## 2. Nutritional Composition

### 2.1. Protein

One of the major biomacromolecules studied in mango leaves is protein. Protein acts as a building block of cell and also plays a major role in growth, maintenance, enzyme regulation, cell signaling, and also acts as biocatalyst [10]. Studies on MLs are limited, considering their role in improving the biomass and their suitability as fodder crop have been investigated in animal models. Studies conducted on MLs meal found crude protein (CP) content (171.4 g kg^−1^ DM) to determine performance, nutrient utilization, and carcass evaluation of growing rabbit [11]. ML protein was assessed for its efficacy as a fodder crop in the meals fed to animals such as rabbit and black Bengal goats [11,12]. ML is a good source of supplementary protein, vitamins, and minerals. These can be utilized as an alternative source of livestock feeding in developing countries for alleviating food shortage for livestock. Proximate composition of MLs showed 13.6% of CP in dauphiné Mauritian variety, 20.38% CP in Nigerian variety, and 6.90% CP in Laos variety [13]. Varietal difference of leaf protein was also studied in five different cultivars of mango (Pusa Arunima, Pusa Surya, Amrapali, Mallika, and Dushehari) grafted on three different rootstocks (K-5, Kurakkan, and Olour), and it was found that Amrapali on K-5 rootstock has higher leaf protein (146.47 mg g^−1^ fresh weight) followed by Amrapali on Olour rootstock (145.22 mg g^−1^ fresh weight). Hence, this study showed a significant effect of rootstocks on protein content in MLs [14].

### 2.2. Lipid/Oil Profile

Mango is known for its pleasant aroma, which varies with the place of origin, variety, and climatic conditions. Hydro distillation method is generally used to extract the essential oil from MLs, and its chemical profile was analyzed by gas chromatography coupled with a mass spectrophotometer. MLO was found to contain monoterpenes (46.98%), sesquiterpenes (38.17%), minor quantities of their analogues (10.67%), and trace amounts of non-terpenoid hydrocarbons and oxygenated hydrocarbons (4.19%). MLO profile showed the presence of seven chemical compounds camphene, α-pinene, α-copaene, pinene, α-gurjunene, β-elemene, and α-humulene [15]. MLO extracted through hydro-distillation method was rich in sesquiterpenes (70.3%) and dominant compounds δ-3-carene (20.5%), α-gurjunene (19.2%), β-selinene (13.9%), and β-caryophyllene (13.7%) [16]. Studies were also conducted to analyze the chemical composition of MLO from different varieties. MLO extracted from Tommy Atkins cultivar showed β-selinene (29.64%), caryophyllene oxide (12.40%), and humulene epoxide II (8.66%) as the main constituent, while MLO from Rosa, Moscatel, and Jasmim cultivars showed caryophyllene oxide and humulene epoxide II as the main constituent. Other constituents that were common in all the four varieties were spathulenol, italicene epoxide, caryphyllene oxide, cyclocolorenone, and humulene epoxide II. This study also reported sesquiterpenoids as the major compound of MLO [17]. The essential oil from MLs also possesses bacteriostatic properties and contains several antimicrobial constituents such as gurjunene, trans-caryophyllene, humulene, selinene, and camphor. However, varietal variation was also found in context of oil composition, where five Egyptian mango cultivars leaves, Alphonso, Sidik, Ewase, Zebda, and Fagrikalan, were evaluated for their antibacterial activities and possibility to be used as food preservatives. The chemical constituents of the MLO were identified using GC–MS spectrometry and showed the presence of several biologically active compounds, such as humulene-4-hydroxy-4-methyl-2-pentanone, elements, and trans-caryophllene, which was common in all five cultivars of mango [18]. In another study, volatile compounds of MLs (*Mangifera indica* var. Coquinho) were extracted using head space solid phase micro extraction (HS-SPME) and hydro-distillation (HD) methods. Gas chromatography–mass spectroscopy (GC–MS) analysis showed a presence of as many as 100 compounds in both leaves and fruit of mango. It was observed that HS-SPME has great potential in isolation of volatiles rich in monoterpenes, whereas these compounds are most likely lost during HD. HD extracts were exclusively detected with oxygenated compounds, viz. carotol, terpineol, cadinol, humulene epoxide II, caryophyllene oxide, and guainol, which may be due to the thermal oxidation of products during distillation. The major compounds found in immature leaves were cyperene, α-humulene, E-caryophyllene, and terpnolene, whereas the main compounds in the mature leaves were cyperene, α-gurjunene, E-caryophyllene, β-cedrene, and α-humulene [19]. The volatilome of MLs demonstrates the presence of very active terpenes, which have health promoting functionalities in the human body. Therefore, volatile compounds can find an important application in the food and nutraceutical industries. The nutritional profile of the MLs, including proteins, components of essential oil, and fatty acid profile, is presented in Table 1.

### 2.3. Minerals and Vitamins

MLs are the potential source of minerals, viz. potassium (K), phosphorus (P), nitrogen (N), calcium (Ca), iron (Fe), sodium (Na), magnesium (Mg), boron (B), zinc (Zn) manganese (Mn), and vitamins, viz. A, B, E, and C. Ali et al. [20] reported concentrations of various minerals in MLs as 589 (K), 480 (P), 343 (Fe), 98 (Mg), 368 (Ca), 28 (Na), 14 (Zn), 3 (Mn), and 2 (N) mg/100 g dry weight (DW). Another study also found Ca (4.41%), Mg (1.58%), K (0.55%), Na (0.23%), N (2.60%), and P (0.40%) in MLs [21]. Various studies reported the concentration of minerals present in MLs as N (0.003–2.6%), P (0.007–0.48), Ca (0.003–4.41%), Mg (0.009–1.58), S (0.37–0.88), Zn (0.0024–0.014%), Na (0.003–0.23%), B (0.0016–0.0042%), copper (0.0021–0.0029%), Fe (0.0062–0.034%), Mn (0.0028–0.003%), and cadmium (0.015%) [13,20,21,22,23,24]. These minerals are important for human nutrition, as they play a major role in various activities such as maintenance of healthy bones and teeth, nerve functioning, muscles contraction and relaxation, immune system health, blood pressure regulation, blood clotting, energy metabolism, and part of many enzymes [25]. The concentrations of vitamin A, B, E, and C reported as 121, 189, 10, and 30 mg/100 mL in ML [20]. In another study, Princwill-Ogbonna et al. [21] found vitamin composition in ML as vitamin A (22.60 mg), B1 (0.48 mg), B2 (0.21 mg), B3 (0.38 mg), and C (13.20 mg). The reported range of various vitamins in MLs was vitamin B1 (0.04–0.48 mg/100 g), B2 (0.06–0.21 mg/100 g), B3 (0.38–2.20 mg/100 g), C (13.20–53 mg/100 g), and A (22.60 mg/100 g) [21,22,24]. These vitamins have numerous health promoting activities in human body viz. development of epithelial tissue, maintaining skin health, visual sharpness, bone development, wounds healing, immune response, and providing strength to the connective tissues. Vitamins B1, B2, and B3 act as a cofactor for various enzymes involved in carbohydrate metabolism and oxidation–reduction reactions [25]. Mineral and vitamin composition of MLs reported by various researchers is presented in Table 2.

## 3. Phytochemical Profile

**Figure 1:**
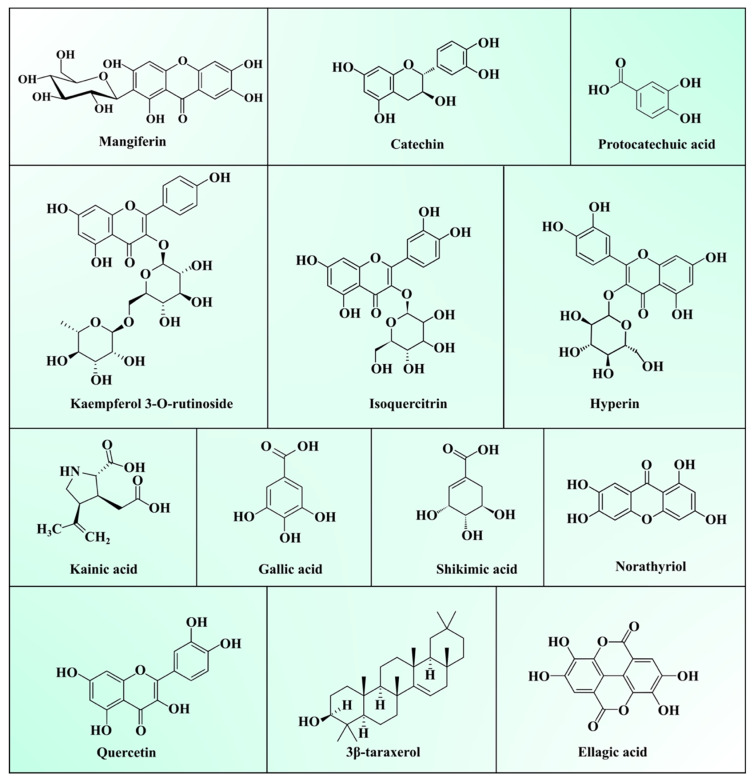
Structure of major compounds present in mango leaves.

Generally, MLs are burnt or discarded, considered as an agricultural crop waste. However, the medicinal properties of MLs make them a useful ingredient in traditional folk tea preparation, and to treat diabetes and respiratory diseases in Asian and African countries [2,26]. As described in the previous Section 2.1, Section 2.2 and Section 2.3, they a contain superior quality of bioactive polysaccharides, proteins, lipids, vitamins, and minerals. Bioactive phytochemicals present in MLs extracts have a high potential in terms of biological and pharmacological activities viz. antioxidant, antidiabetic, anti-inflammatory, antimicrobial, antiviral, immunomodulatory, anti-obesity, antiallergic, antifungal, antiparasitic, antidiarrheal, antipyretic, and anti-tumour activities [9,27]. Phytochemicals present in MLs can be broadly categorized as polyphenols, terpenoids, carbohydrates, sterols, carotenoids, vitamins, fatty acids, and amino acids. Among them, total phenolic compounds (TPC), including phenolic acids, xanthones, benzophenones, tannins, terpenoids, and flavonoids, are most abundant in ML. Several epidemiological studies have proved the activities of TPC against chronic diseases viz. cancer, diabetes, and cardiovascular and neurodegenerative diseases [28]. TPC modulates numerous physiological processes such as enzymatic activity, cell proliferation, signal transduction pathways, and cellular redox potential to fight against chronic pathologies [29]. A list of various phytochemicals present in ML is depicted in Table 3, and structures are presented in Figure 1.

TPCs possess an aromatic benzenoid-ring attached to a hydroxyl group. Among all TPCs, mangiferin particularly is a potential bioactive compound in ML. In recent years, diets enriched with bioactive compounds are getting much attention due to their potential to minimize the risks of several chronic diseases’ development. Pan et al. [30] stated mangiferin (7.43%) is a major constituent in ML extract, whereas other compounds reported in higher concentration include quercetin-3-O-β-Dglucoside (0.82%), quercetin-3-O-β-D-galactoside (0.86%), and isoswertisin (1.25%). Mangiferin is a natural xanthonoid polyphenol antioxidant and plays role in ameliorating insulin resistance, modulating glucose metabolism, lowering cholesterol synthesis, and inhibiting the expression of the inducible nitric oxide synthase and TNFα [34]. TPC and total flavonoid content in crude, MeOH, and EtOAc extracts of ML was reported as 230, 99, and 186, and 131, 46, and 191 mg/g, respectively. The phytochemical investigation of MeOH, EtOAC, and crude extracts of ML using ultra high-pressure liquid chromatography (UPLC)-MS/MS identified several secondary metabolites, including eleven phenols, ten benzophenones, nine flavanols, four derivatives of gallotannins, four xanthones, and seven terpenoids. The EtOAC extract showed higher TPC and total flavonoids (TFC) compared to the MeOH extract [13]. Ouf et al. [18] identified 83 compounds in ML essential oils of five cultivars using gas chromatography–mass spectrometry (GC–MS). Among them, trans-caryophyllene (8.06–18.88%), α-selinene (4.33–16.92%), and α-humulene (8.48–25.98%) were found in the higher concentrations. Gu et al. [31] isolated and characterized four benzophenone derivatives, manindicin A, manindicin B, mangiferin, and norathyriol from ML extract by nuclear magnetic resonance (NMR) spectroscopic technique. Some of these compounds exhibited significant *α*-glucosidase inhibitory, immunosuppressive, and antioxidant activities. These studies proved that ML can be served as potential source of food supplement for improving human health.

## 4. Biological Activities of the Mango Leaves Extract

### 4.1. Anticancer Activities

Cancer is one of the most prevalent global threats after cardiovascular disease. Thus, there is an imperative need to undertake novel treatment strategies to counter this global issue. Polyphenols present in MLs like gallotannins, phenolic acids, quercetin, and mangiferin exhibit chemo-preventive effects against various cancer types due to their anti-inflammatory and antioxidant effects [35]. Antitumoral activities of MLs extract are mainly attributed to the primary bioactive xanthone glucoside, mangiferin. These compounds are demonstrated to suppress several cancers by impeding their invasion, migration, and proliferation [36]. Mangiferin is found to overturn the transition from epithelial-to-mesenchymal in MCF7 breast cancer cell lines by inhibiting Wnt/β-catenin pathway and by downregulating the expression of specific enzymes (5′Nucleotidase, γ-GT, and aryl hydrocarbon hydroxylase) in lung cancer bearing albino mice [37,38]. It also suppresses the levels of Akt phosphorylation and cyclin B1, causing spontaneous cell cycle arrest in G_2_/M phase. It was also found to instigate Nrf2-mediated antioxidant activities at a concentration of 50 μM, with no influence of myeloid leukaemia cell sensitivity to chemotherapeutics [39]. Mangiferin was also found to mitigate the oxidative stress and inhibit methylmercury-induced DNA damage in human neuroblastoma cell line IMR-32 [40]. The anti-invasive and antimetastatic activities of mangiferin could also be attributed to its ability to regulate the expression of metalloproteinases, which determines the cell proliferation and inhibits epithelial–mesenchymal transition, eventually causing a loss in cell adhesion.

**Figure 2:**
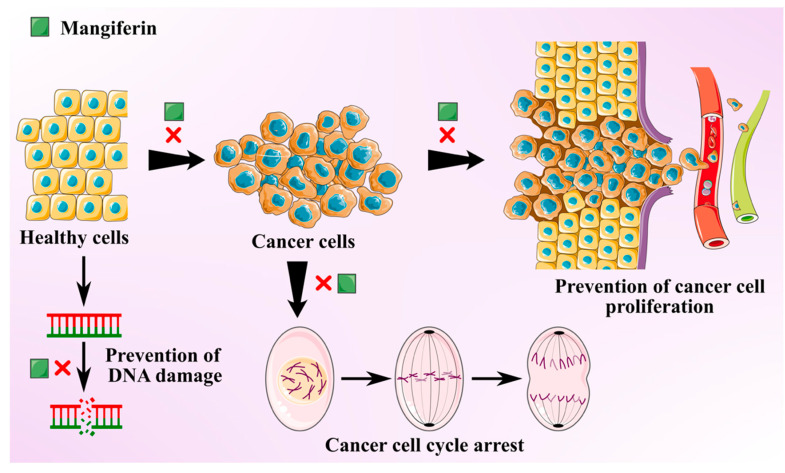
Schematic showing anti-cancer activity of the mangiferin from mango leaves.

A study was conducted to investigate the antitumoral effects of ML extracts on (MDA-MB-231) highly and (MCF7) minimally invasive breast cancer cells and (MCF10) non-tumorigenic cells at IC_50_ >200 µg/mL [41]. The leaf extracts displayed protective properties against cytotoxic and oxidation effects on breast cancer cell lines and minimal damage to non-carcinogenic cells. MLs extracts with a high concentration of homo-mangiferin and methyl gallate were found more effective against MDA-MB-231 cells, while gallotannins showed cytotoxicity against MCF7 cells. In another study, ethanolic extract of mango leaves at a concentration of IC_50_ >200 µg/mL exhibited cytotoxic activities against lung fibroblast (ATCC CLS 300421,WI-38 VA-13 subline 2RA), skin fibroblast (ATCC CRL1947, CCD-986SK), colon adenocarcinoma (ATCC CCL227, SW 620), gastric carcinoma (ATCC HTB103, Kato-III), liver hepatoblastoma (ATCC HB8065, Hep-G2), bronchogenic carcinoma (ATCC HTB-168TB, Chago K-1), and ductal carcinoma (ATCC HTB20, BT474) [42]. Similarly, MLs extract was used to synthesize silver nanorods. These nanorods exhibited strong in vitro cytotoxicity, antioxidant, and anticancer activities at 10% *w/v* against colorectal carcinoma and breast cancer cell lines (HCT-116, MCF-7) [43]. Anti-cancer potential of the mangiferin is schematically depicted in Figure 2.

### 4.2. Anti-Diabetic Activity

Diabetes is a chronic metabolic disorder that badly disturbs the health and quality of human life and is established as the foremost threat to society irrespective of geographical locations. Diabetes is characterized by elevated glucose or above-normal glucose level (70–110 mg/dL), which are partially due to oxidative damage to pancreatic β-cells, leading to a decline in insulin secretion [44]. Insulin regulates the blood glucose level (BGL); low secretion of insulin causes hyperglycemia, which enhances oxidative stresses and eventually causes several health problems like frequent urination, thirst, and hunger [45]. In 2016, the International Diabetes Federation (IDF) reported that around 415 million people are diabetic, with a population of 642 million predicted to suffer from type-2 diabetes (diabetes mellitus or DM) by 2040 [46]. Several medicines such as acarbose are currently used in diabetes, but such types of diabetic medicines lack DM restraint and revealed undesirable side effects over time [45]. All over the world, researchers are exploring medicinal plants as an effective way to cure this debilitating disorder, because medicinal plants are a rich source of bioactive constituents, and most of them are known to be potent against DM.

**Figure 3:**
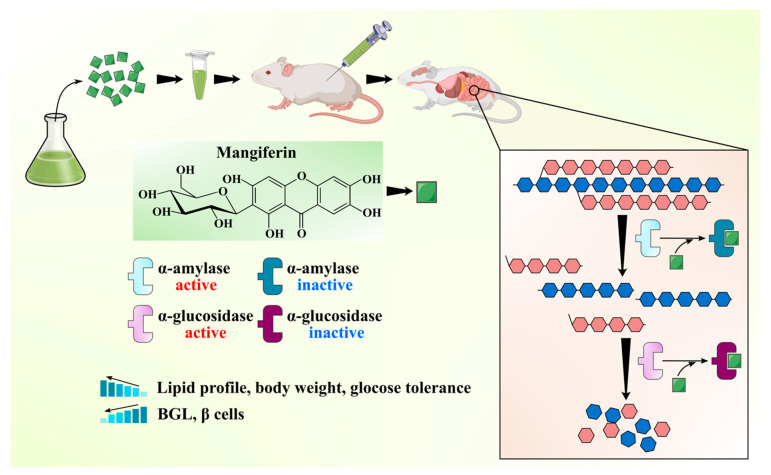
Schematic showing anti-diabetic activity of the mangiferin from mango leaves.

MLs have been widely claimed as effective ethnomedicine against DM due to their anti-diabetic bioactive constituents like benzophenones (mangiferin) and flavonoids (quercetin and its glucoside forms). One of the best effective approaches in the cure of DM is the inhibition of α-amylase and α-glucosidase enzymes, which regulate postprandial glucose absorption [47]. A comparative analysis of mangiferin and MLs extract was done to check the efficiency of each extract to inhibit α-glucosidase enzymes. MLs extracts at a concentration of 100, 250, and 500 mg/mL caused up to 77.8%, 83.4%, and 95.7% inhibition of α-glucosidase, respectively. At the same time, mangiferin at a concentration of 10, 25, and 50 resulted in 86.85%, 92.35%, and 99.11% inhibition of α-glucosidase, respectively. It can be inferred that mangiferin is an active ingredient in the inhibition of α-glucosidase enzyme activity and in managing the diabetic condition [48]. Ganogpichayagrai et al. [42] evaluated the anti-diabetic activity of mangiferin and MLs extract through the inhibition of α-glucosidase and α-amylase in vitro. Authors reported that mangiferin showed strong inhibition of rat α-glucosidase with a median inhibitory concentration IC_50_ of 433.3 µg/mL and MLs extract showed potent inhibition of yeast α-glucosidase with the IC_50_ of 50.3 µg/mL. Saleem et al. [49] evaluated the anti-diabetic potential of MLs extract (550, 750, 950 mg/kg) cv. Ratol in alloxan monohydrate (150 mg/kg) induced diabetes in albino mice. Authors found that the administration of MLs extract in chemically induced diabetic mice reduced the postprandial glucose level, prevented the surge of glucose in the blood, and improved the lipid profile along with body weight. Different bioactive compounds have been isolated from the MLs extract and demonstrated their anti-diabetic potential. Gu et al. [31] isolated and characterized four bioactive compounds as manindicins A and B, mangiferin, and norathyriol (deglycosylated mangiferin) from MLs extract. Authors revealed that norathyriol exhibited strong α-glucosidase inhibition with IC_50_ of 4.22 ± 0.19 μg/mL, which was four-fold effective with respect to commercial inhibitor acarbose (IC_50_: 16.28 ± 1.22 μg/mL), while mangiferin (IC_50_: 32.11 ± 2.01 μg/mL) and manindicin A (IC_50_: >300 μg/mL) and B (>300 μg/mL) displayed weaker α-glucosidase inhibition. The less inhibitory potential of Mangiferin may be due to its molecular size and polarity. The replacement of glucose moiety with hydrogen may weaken the steric hindrance during mangiferin–enzyme interaction and enhance the inhibitory potential of mangiferin towards α-glucosidase [31]. The anti-diabetic potential of the mangiferin is schematically shown in Figure 3. The anti-diabetic potential of mangiferin was also demonstrated, as it increases insulin sensitivity and inhibits α-glucosidase [50]. Similarly, for quercetin, α-glucosidase inhibition was substantially higher than that of its 3-O-glucoside hyperoside [51]. Bhuvaneshwari et al. [52] investigated the anti-diabetic activity of tender and mature leaves of totapuri cultivar of mango, and authors found that tender leaves extract (500 mg/kg) efficiently inhibited the α-amylase with IC_50_ 22.01 µg/mL, while mature leaf extract (500 mg/kg) exhibited the α-glucosidase inhibition with IC_50_ 21.03 µg/mL. Findings suggest that bioactive compounds from the ML can be effective in reducing the risk of diabetes.

### 4.3. Antioxidant Activities

Many recent studies have shown that free radicals generated during the metabolic process contribute to various degenerative diseases such as acquired immunodeficiency syndrome, ischaemic diseases, neurological disorders, and many more [53]. Antioxidant substances, on the other side, provide a high level of antioxidant activity to lessen the adverse effects of free radicals. MLs were reported to have antioxidant capacity due to the presence of phenolics and flavonoids in different studies [54]. High-performance liquid chromatography coupled to electrospray ionization and quadrupole time-of-flight mass spectrometry (HPLC-ESI-qTOF-MS/MS) analysis of MLs extract had identified neomangiferin, mangiferin, kaempferol-3-O-rutinoside, isoquercitrin, and quercetin as the main compounds and also reported that these compounds contributed directly to the antioxidant activity of MLs [55]. The 2,2-diphenyl-1-picrylhydrazyl (DPPH) assay and superoxide dismutase (SOD)-like activity had shown that MLs serve as a moderate antioxidant with an IC_50_ value of ~9 and 117 μg/mL [56]. In another analysis, MLs methanol extract provided radical scavenging activity with an IC_50_ value of 13.37 μg/mL [57]. Fraction analysis of MLs extract with n-butanol, hexane, and ethylacetate demonstrated that ethylacetate fraction had the highest antioxidant capacity of 1226 and 2817.99 mg trilox/g estimated using DPPH and 2,2′-azino-bis-3-ethylbenzthiazoline-6-sulphonic acid (ABTS) assay, respectively, and reducing power of 10172.59 µmol FeSO_4_.7H_2_O/g extracts analyzed through ferric reducing antioxidant power (FRAP) assay [58]. Similarly, subcritical water extracts of MLs had antioxidant activity index (AAI) values of 7.92 ± 0.16 and demonstrated superior activity to (+)-α-tocopherol (AAI = 3.65 ± 0.07) [41]. Trolox equivalent antioxidant ability (TEAC) study of MLs extracts had recorded 2.13 and 2.59 mmol TE/g DW TEAC values, respectively, for mangiferin pentoside and benzophenones [59]. The efficacy of the MLs was also studied in the chitosan-based films, and it was found that antioxidant capacity of the MLs supplemented chitosan films improved in a dose-dependent manner [60]. The antioxidant activity of hydroalcoholic MLs extract fermented with either *Lactobacillus casei* or effective microorganisms had higher antioxidant activity. The study also showed that fermented extracts decreased lipopolysaccharide-generated reactive oxygen species [61]. In an advanced study, MLs extract was found suitable as a green antioxidant for increasing the storage life of biodiesel [62]. In summary, many interesting results indicated the potential of MLs extract as an antioxidant with wider applicability in food, food packaging, and many more industries.

### 4.4. Antimicrobial Activities

**Figure 4:**
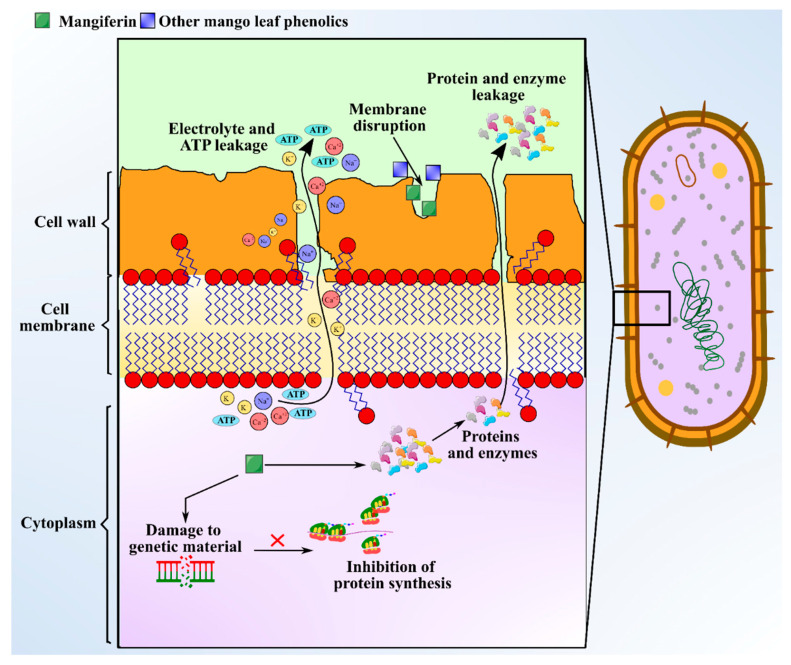
Schematic showing anti-microbial activity of the mangiferin from mango leaves.

There is immense interest in unravelling the role of bioactive compounds present in nature. Some medicinal plants with antimicrobial attributes are capable of evading the activity of multi-drug resistant (MDR) microbes, which helps in withstanding antimicrobial resistance [63]. Distinct morphological parts of the mango plant like leaves, stem, kernel, seeds, and bark have been manifested to show antimicrobial activities against microbes like *Staphylococcus* sp., *Bacillus subtilis, Escherichia coli, Candida albicans, Proteus vulgaris, Pseudomonas fluorescens, Shigella flexneri, Klebsiella pneumoniae,* and *Salmonella typhi*. MLs extract is the most studied part for antibacterial effects. Bharti [64] observed that hexane and hexane/ethyl acetate extracts of MLs exhibit favorable antibacterial effects against *Mycobacterium tuberculosis* and *Enterobacter aerogenes*. Antimicrobial investigation of the essential oils extracted from leaves of five Egyptian mango cultivars to be used as preservatives materials has been demonstrated by Ouf et al. [18] against *Staphylococcus* sp. (Minimum Inhibitory Concentration (MIC): 62.5 μg/mL), Bacillus subtilis (MIC: 125 μg/mL), *Escherichia coli* (MIC: 125 μg/mL), *Pseudomonas aeruginosa* (MIC: 500 μg/mL), Aspergillus flavus (MIC: 1000 μg/mL), and Salmonella typhi (MIC: 1000 μg/mL). The major phytochemicals responsible for the antimicrobial activity in mango leaves include phenolics, alkaloids, saponins, glycosides, terpenes, and tannins. The concentration of the aforementioned compounds were measured as follows: flavonoid content was the highest at 11.25 mg/100 g; there was 3.23 mg/100 g of saponins; phenolic content was 0.08 mg/100 g; and tannins in leaves was at 0.46 mg/100 g [24]. Polyphenols and phenolic acids present in ML extract include protocatechuic acid, gallic acid, hyperin, catechin, quercetin, kainic acid, ethyl digallate, ellagic acid, and shikimic acid, which can inhibit the growth of pathogens [8]. The mechanism of exertion of antimicrobial activity by these compounds involves depleting intracellular ATP levels, depolarization of plasma membrane, cytoplasm leakage, damaging genetic material, and declining the concentration of microbial protein [65]. Anti-microbial activity of mangiferin is schematized in Figure 4. Additionally, an adequate level of antibacterial activity of leaf extract was found against Gram positive bacteria, but no or less activity against Gram-negative bacteria was observed [66]. The study indicated that MLs extract exhibited diameter of zone of inhibition in the range of 7.0‒11.5 mm against Gram-positive bacteria like *Staphylococcus* and *Bacillus* sp., but no activity was seen against gram-negative *Salmonella* spp. Additionally, Mangiferin, a xanthone C-glycosyl compound extracted from MLs extract, has also shown to possess strong iron chelating activity, which favors antimicrobial activity. Furthermore, chemical analysis of MLs extract for antimicrobial activity indicated the presence of five major flavonoid compounds including epicatechin-3-O-β-glucopyranoside, 5-hydroxy-3-(4-hydroxyl phenyl) pyrano chromene-4 (8H)-one, 6-(phydroxybenzyl) taxifolin-7-O-β-D-glucoside, quercetin-3-O-α-glucopyranosyl-(1-2)-β-D-glucopyranoside, and epicatechin(2-(3,4dihydroxyphenyl)-3,4-dihydro-2H-chromene-3,5,7-triol [67]. These compounds are identified to be synthesized immediately after a fungal attack, and a concentration of 1000 ppm has curtailed the growth of target fungal species like *Aspergillus* and *Alternaria* from 56‒97% [67]. A myriad of terpenes identified from MLs extract including α-pinene, β-pinene, δ-elemene, taraxerol, β-elemene, camphene, γ–cadinene, lupeol, friedelin, linalool, α-guaiene, humulene, α-farnesene, myrcene, limonene, β-ocimene, γ–terpinene, and α-terpinolene exhibit bacteriostatic and bactericidal effects against different pathogens [8]. HPLC-TOF-ESI/MS analysis of leaf extract to identify hydrolyzable tannins revealed the presence of gallatotannins. Antimicrobial properties of gallatotannins have been associated with their ability to hinder the enzymes of the pathogen, disintegrate lipid bilayer membranes, and promote chelation of metal ions [68]. The MIC of MLs extract is reported to be in the range 11–52 mg/mL [69] and the MIC values were greatest against *E. coli* with 36.3 mg/mL. The aforementioned phytocompounds purified from ML extract can be directly used as food additives to enhance the shelf-life of foods, and as an alternative for the synthetic antimicrobial agents, owing to their broad biological and pharmacological activities [70].

### 4.5. Hepatoprotective Properties

Hepato steatosis or fatty liver disease (FLD) is mainly caused by an imbalance in production and metabolization of fat in the body. Higher consumption of fat also negatively affects oxidation of fatty acid in the liver and enhanced deposition in the chief functional cells of the liver (hepatocytes), which causes oxidative stress and hepatic steatosis. A very high fat cell deposition alters the signaling pathways. The peroxisome proliferator-activated receptor (PPAR) and nuclear factor kappa B (NF-kB) pathways are involved in the obesity inflammation process. The progression of liver damage may result in subclinical icteric hepatitis to necro-inflammatory hepatitis, cirrhosis, and carcinoma [71]. Therefore, the compounds having a role as an antioxidant and inhibitor of lipid peroxidation and their free radical scavenging capacity may show the hepatoprotective properties. MLs tea was prepared using Ubá variety leaves [72]. This MLs tea contains about 0.72 mg/mL mangiferin, 1.59 mg GAE/mL total phenolic content, 80% of radical scavenging activity, and 0.007 µg/µL EC_50_ value. The 25 mL/day dose of MLs tea was given to fat obese rats and the effects were evaluated. The rats consuming a high-fat diet activated hepatic steatosis and increased accumulation of cell fat by 46% compared to control. Treatment of rats consuming a high fat diet with MLs tea increases the nucleus, cytoplasm blood vessels percentages, and reduced accumulation of fat droplets, consequently reducing the incidence of FLD. These hepatoprotective activities of MLs tea were mainly due to gene expression. It is reported that increased expression of PPAR-α enhances oxidative stress, inflammation, and lipid metabolism in obese mice, whereas negative modulation of PPAR-α and adiponectin receptor II (AdipoR2) results in FLD [73]. High fat diet decreases the expression of mRNA of PPAR-α and AdipoR2, while MLs tea up-regulates the gene expression of PPAR-α and AdipoR2, attenuating FLD. At the same time, the expressions of NF-κB p65 and sterol regulatory element-binding proteins (SREBP1c) genes were reduced in cells of liver. It was also concluded that MLs tea improves the situation of FLD by acting as an anti-inflammatory agent via PPAR-α activation and NF-κB modulation [74]. Therefore, MLs products can be used as hepatoprotective and alternative for the treatment of fatty diet induced FLD or hepatic steatosis.

### 4.6. Anti-Obesity and Lipid Lowering Activity

Obesity is the one of the most prevalent disorders in the world due to dietary habit, sedentary lifestyle, and stress, which promotes various cardiovascular disease and pathological conditions like thrombosis atherosclerosis, hypertension, inflammation, and hepatosteatosis. These problems can be overcome by a change in lifestyle and/or by using drugs. However, there is a need for an alternative natural way for treatment due to numerous side-effects of available drugs. Hypocholesterol activity of methanolic extract of MLs was evaluated using in-vitro pancreatic cholesterol esterase inhibition assay to identify bioactive compounds related to it. The study revealed that the mangiferin is the major compound present in MLs extract, but does not alter the cholesterol esterase inhibition assay, whereas 3b-taraxerol (IC_50_ value of 0.86 μg/mL) exhibited hypocholesterol activity [75]. In an independent study, the effect of MLs extracts from Ubá variety was evaluated for the anti-obesity activity in obese rats (male Wistar rats) fed with high-fat diet. The consumption of MLs tea at the concentration of 24.7 mL/day resulted in increased antioxidant activity along with anti-inflammatory effects. The finding was established by increased total antioxidant activity and concentration of interleukin10, decreased abdominal fat accumulation, increased expression of PPAR-γ, and lipoprotein lipase and reduction in the expression of fatty acid synthase [76]. Authors concluded that MLs tea have anti-obesity therapeutic potential by modulating the expression of enzymes and transcriptional factors related to adipogenesis. In in-vivo condition, cholesterol lowering activity was evaluated in female albino Wistar rats. Three major compounds, viz. 3β-taraxerol, mangiferin, and iriflophenone-3-C-β-glucoside, were identified through HPLC. The plasma triglycerides were significantly reduced by oral dose of MLs extract (90 mg/kg from day 21 to 42), which confirms cholesterol-lowering activity of MLs extract [77]. Sandoval-Gallegos et al. [78] prepared a methanolic extract and evaluated it in vitro and in vivo. In the experiment, a dyslipidemia model using Wistar rats was practised to evaluate the effect of MLs extract on lipid levels in the blood. Rats were treated with 100, 200, or 400 mg/kg MLE and evaluated at the zeroth, 15th, and 32th day. Studies showed the application of MLs extract lowered the cholesterol by 40–47% and exhibited about 62% antihyperlipidemic activity compared to control at an oral dose of 200 mg/kg. It also maintains lower triglycerides level and enhanced high-density lipoprotein (HDL) cholesterol by 2.44 and 4.11 times at the 15th day in MLs extract treated rats (200 and 400 mg/kg, respectively). The above-mentioned studies showed the potential of ML in lowering the body fat and reducing the occurrence of cardiovascular disease related to obesity. The biological activities of MLs extract is reported in Table 4.

### 4.7. Anti-Diarrheal Activity

Diarrhea is one of the most infectious diseases, caused due to drinking of unsafe water, a situation of poor sanitation and hygiene, uncooked meat, and food intolerances, which leads to 3.2% of mortalities globally [79,80]. The key organisms responsible for this disease include microbial communities like *Escherichia coli, Candida albicans, Vibrio cholerae, Shigella flexneri, Staphylococcus aureus,* and *Salmonella typhi*. Diarrhea accounts for about 1.6 million deaths as per reports of WHO generally observed in developing countries, causing 28% mortality in infants residing in Africa and Southeast Asia due to serious gastroenteritis [81,82]. Therefore, researchers throughout the globe are paying attention to the search for new plant-based therapeutic agents that can overcome this global problem [83]. Recently available medicines are having various side effects with some toxicity. The occurrence of diarrhea in developing nations is facing scarcity of traditional antidiarrheal medicines and healthcare facilities. Hence, conventional drugs from medicinal plants are the only solution control to diarrhea in such countries [84]. De et al. [85] reported effects of aqueous young leaves extract of mango on Gram-negative microorganisms causing gastrointestinal disorders. The authors reported that phytochemicals present in the crude extract play a vital role as antidiarrheal agent. Aqueous extract of MLs was screened against various pathogens like *E. coli, S. typhi, Vibrio cholera,* and *S. sonnei* at a dose level of 300, 200, 100, and 50 mg/mL. It was observed that the antidiarrheal activity increased with an increase in dose level. Hence, it was concluded that the aqueous young leaves extract of mango plant can treat the situation of diarrhea.

Yakubu et al. [86] reported antidiarrheal activity of aqueous extract of MLs in female albino rats. They observed that the leaves sample was rich in phytochemicals like flavonoids, saponins, phenolics, and alkaloids. The dose level of 25 and 50 mg/kg body weight reduced the total quantity of wet feces and enhanced the reticence of evacuations. MLs extract demonstrated the most visible antidiarrheal activity at a concentration of 100 mg/kg of body weight, where the situation of diarrhea was prevented exponentially, with an increase in dose level reduced the multitudes and capacity of intestinal fluid. Ricinoleic acid from castor oil has been utilized as a diarrhea stimulating agent in various studies on rat models. Ricinoleic acid inhibits the activity of Na^+^ –K^+^ ATPase in the gut and reduces the active absorption of Na^+^ and K^+^. Extracts at different concentrations tremendously enhanced the Na^+^-K^+^ ATPase potential in the small intestine, which is one of the mechanisms of MLs extracts to act as an antidiarrheal agent.

## 5. Toxicological/Allergenicity Evaluation of MLs

Numerous studies have pointed out the various beneficial effects of mango leaves extract against cancer, diabetes, cardiovascular, and neurodegenerative diseases. These positive effects are due to the presence of a plethora of phytochemicals such as mangiferin followed by phenolic acids, benzophenones, and other antioxidants such as flavonoids, ascorbic acid, carotenoids, and tocopherols (20). Few studies also reported the toxic effects of MLE attributed to the presence of allergens. Mango allergy can occur in two ways: either the immediate hypersensitivity reaction as wheezing dyspnoea, anaphylaxis, erythema, urticaria, and angioedema, or the late reaction presenting as contact dermatitis and periorbital oedema [93]. Transcriptome analysis using high-throughput llumina sequencing of leaf and fruit reported the presence of 66 potential allergen genes mainly belonging to pollen allergen, pathogenesis-related protein Bet v I family and NADPH-dependent flavin mononucleotide (FMN) reductase [94]. A long-term study (three consecutive months) on Sprague Dawley rats at various doses (100, 300, and 900 mg/kg) of MLs revealed the slight body weight increase and higher fat weight, the serum thyroglobulin and cholesterol levels, and the slight increase in epididymis weight of male rats compared with the control group. The study also revealed no abnormalities on short-term exposure (14 days). The study concluded that MLs extract was safe at the maximum dose of 18.4 g/kg weight of mice [95]. In an independent study, MLs extract containing 60% mangiferin was evaluated in Han:Wist male and female rats in a 90 day study, and it was concluded that ML extracts demonstrated no observed adverse effect at the highest dose tested (2000 mg/kg body weight per day) [96]. Urushiol, a hapten to skin proteins that induces a type IV hypersensitivity response, is an allergenic substance found in the Anacardiaceae family, most commonly known for poison ivy (*Toxicodendron radicans*) and poison oak (*T. diversilobum*). Hershko et al. [97] reported the fact the individual with prior exposure to poison ivy/oak allergy may develop allergic contact dermatitis from mango on first exposure. Cross-sensitization of these plants with mango is known to be secondary to a superposition of urushiol antigen and 5-resorcinol, primarily found in mango peels [98]. These studies overall suggest that mango has some allergic responses, but those reactions have been limited to mango latex, pollen allergen, or prior urushiol exposure [99].

## 6. Conclusions

MLs exhibit exceptional biological, medicinal, and metabolic properties. MLs are otherwise considered as a waste material generated mainly through the pruning of mango plants; in reality, they are a most significant resource containing a wide variety of bioactive compounds (phenolics and essential oils), crude protein, dietary fiber, minerals, and vitamins. The various bioactive compounds present in the MLs include phenolic compounds, flavonoids, benzophenones, sesquiterpenes, saponins, xanthones, tannins, terpenoids, and alkaloids. MLs extract is found useful for the treatment of common diseases like diarrhea, chronic ailments like diabetes, and fatty liver disease. MLs extract possessed strong anti-proliferative activity against pancreatic, breast, human colon carcinoma, and other types of cancers. The extracts of MLs are also crucial in as an anti-obesity agent and demonstrate hepatoprotective action. There are numerous vital chemical compounds present in mango leaves, which are instrumental to performing various metabolic, bacteriostatic, and antimicrobial activities. Some of these include gurjunene, trans-caryophyllene, humulene, selinene, and so on. Mangiferin present in MLs was also found to mitigate the oxidative stress involved in the treatment of numerous pathologies. MLs are thus a potential source of cost-effective food supplements and nutritive ingredients for improving human health and curing acute and chronic diseases.

## Figures and Tables

**Table 1:** Nutritional profile (protein and fatty acid composition) and essential oil components of mango leaves.

Nutritional Component		Composition					References
Protein		g/kg of DM (dry matter)					
Crude protein		93.2					[12]
Crude protein		171.4					[11]
Lipids/oil profile		(%)					[16]
α-pinene		3.0					
δ-3-carene		20.5					
α-gurjunene		19.2					
β-caryophyllene		13.7					
β-selinene		13.9					
Viridiflorene		6.1					
Grouped constituents		(%)					
Monoterpene hydrocarbons		29.2					
Sesquiterpene hydrocarbons		68.2					
Oxygenated sesquiterpenes		2.1					
Total		99.5					
Variety wise fatty acid profile	Composition (%)						[16]
	Ewase	Alphonso	Sidik	Zebda	Fagri-kalan		
Myristic acid	1.74	-	-	15.62	1.13		
Palmitic acid	27.23	1.05	-	56.82	-		
Stearic acid	9.02	1.33	3.67	11.77	2.11		
Oleic acid	13.65	1.45	14.68	7.14	1.04		
Linoleic acid	4.39	1.23	7.25	-	2.70		
Linolenic acid	10.29	22.75	12.48	-	26.43		
Behenic acid	33.68	72.19	50.02	8.65	66.60		
Variety wise chemical composition of essential oil		Composition (%)					[16]
		Tommy Atkins	Rosa	Moscatel	Jasmim		
β-Selinene		29.49	-	-	2.3		
Italicene epoxide		7.81	2.56	4.42	3.32		
Espathulenol		1.93	4.32	9.19	5.81		
Caryophyllene oxide		12.40	23.62	48.42	30.77		
Humulene epoxide II		8.66	11.56	23.45	16.27		
Ciclocolorenone		7.26	5.91	4.55	2.68		
Variety wise chemical composition of essential oil	Composition (%)						[16]
	Ngowe	Apple	Keit	Boribo	Tommy Atkins	Van Dyke	
α-pinene	5.8	10.3	2.4	10.9	24.5	18.0	
Camphene	0.3	0.8	0.5	1.7	0.5	0.8	
β-pinene	7.7	6.8	1.9	21.9	2.9	4.3	
δ-3- Carene	-	-	19.4	-	29.2	17.9	
α-Copaene	1.8	1.5	4.9	1.2	1.4	1.5	
β-Elemene	1.4	1.5	4.1	1.6	0.4	0.9	
α- Gurjunene	4.2	9.7	17..4	8.7	10.3	16.7	
α-Humulene	3.4	2.6	2.7	2.5	1.3	3.9	

**Table 2:** Mineral and vitamin composition of mango leaves.

Group	Composition	References
Mineral	(%)	
Nitrogen (N)	0.003–2.60	[20,21,23]
Phosphorus (P)	0.007–0.48	[13,20,21,22,23,24]
Potassium (K)	0.008–0.95	[20,21,23,24]
Calcium (Ca)	0.003–4.41	[13,20,21,22,23,24]
Magnesium (Mg)	0.009–1.58	[20,21,23,24]
Sulphur (S)	0.37–0.88	[23]
Zinc (Zn)	0.0024–0.014	[20,23,24]
Sodium (Na)	0.003–0.23	[21,24]
Boron (B)	0.0016–0.0042	[23]
Copper (Cu)	0.0021–0.0029	[23,24]
Iron (Fe)	0.0062–0.034	[20,22,23]
Manganese (Mn)	0.0028–0.003	[20,23,24]
Cadmium (Cd)	0.015	[24]
Vitamin	(mg/100 g)	
Thiamine (B1)	0.04–0.48	[21,22,24]
Riboflavin (B2)	0.06–0.21	
Niacin (B3)	0.38–2.20	
Ascorbic acid (C)	13.20–53	
Vitamin A	22.60	[21]

**Table 3:** Phytochemical profile of the mango leaves.

Variety	Type of Extract	Bioactive Compounds Identified	References
Mango leaves	Crude, Methanol, Hexane, Ethyl acetate	Phenolic compounds (gallic acid; derivative of gallic acid; sodium gallate; ellagic acid; protacatechuic acid; methyl gallate; theogallin; derivative of theogallin; tetrahydroxy sodium benzoate), Xanthones (mangiferin; isomangiferin; mangiferin-6′-O-gallate; mangiferin 3-methyl ether), Flavonols (kaemferol; quercetin; quercetin 3-O-glucoside; quercetin pentoside; quercetin carboxylic acid; epicatechin gallate hexamalonate; quercetin 3-O-rhamnoside; rhamnetin; rhamnetin hexoside), Benzophenones [3-glucosylmaclurin; maclurin 3-C-β-D-glucoside, maclurin di-O-galloylglucoside, maclurin 3-C-(6′-O-phydroxybenzoyl)β-D-glucoside, maclurin mono-O-galloylglucoside, maclurin, iriflophenone tri-O-galloylglucoside; iriflophenone 3-C-β-D-glucopyranoside; maclurin 3-C-(6″-O-p-hydroxybenzoyl)β-D-glucoside; iriflophenone-di-O-galloyl glucoside; iriflophenone glucoside derivative], Terpenoids (3,27-dihydroxycycloart-24-en-26-oic acid; 3β-cycloartane-3,29-diol; cycloartane-3,24,25-triol; mangiferonic acid; lupeol; cycloart-25-ene-3,24,27-triol; manglanostenoic acid), Gallotannins (digalloyl glucoside;tri-O-galloyl glucoside; tetra-O-galloyl glucoside; pentagalloyl glucose), Other compound (ferulic acid hexoside)	[13]
Mango leaves	70% ethanol exact	Gallic acid; quercetin; protocatechuic acid; mangiferin; isovitexin; vitexin; Iriflophene; isoswertisin; taxifolin; amentoflavone; hypericin; 2,4,4′,6-tetrahydroxybenzophenone-3-β-D-glucoside; gvajaverin; 4′,6-dihydroxy-4-methoxybenzophenone-2-O-β-D-glucoside; 2,4′,6-trihydroxy-4- methoxybenzophenone-3-C-β-D-glucopyranoside; hyperoside; 2,4,4′,6-tetrahydroxybenzophenone-3-C-(2-O-p-hydroxybenzoyl-p-hydroxybenzoyl)-β-D-glucoside; methyl-2-O-β-D-glucopyranosylbenzoate; foliamangiferoside A1; isoquercitrin; 4′,6-dihydroxy-4-methoxybenzophenone-2-O-(2″),3-C-(1″)-1″-desoxy-β-fructopyranoside; quercitrin; quercetin-3-O-β-D-xylopyranoside; quercetin-4′-O-β-D-glucoside; 3′,5′-dimethoxy-4′,5,7-trihydroxyflavone; 4′-O-p-hydroxybenzoylmangiferin; 2,4′,6-trihydroxy-4-methoxybenzophenone-3-C-(2-O-p-hydroxybenzoyl-p-hydroxybenzoyl)-α-D-galactoside; 4,4′,6-trihydroxybenzophenone-2-O-(2″),3-C-(1″)-1″-desoxy-β-fructofuranoside; luteolin-7-O-β-D-glucoside; 4,4′,6-trihydroxybenzophenone-2-O-(2″),3-C-(1″)-1″-desoxy-β-fructopyranoside; 4′,6-dihydroxy-4-methoxybenzophenone-2-O-(2″),3-C-(1″)-1″-desoxy-β-fructopyranoside	[30]
Mango leaves	Petroleum ether, hydro-distilled using a Likens–Nickerson apparatus.	Dodecane; docosane; tetradecane; pentadecane, hexadecane; heneicosane; heptadecane; palmitic acid; nonadecane; eicosane; stigmasterol; squalene; 7-dehydrocholesterol; cholesterol; octadecane; myristic acid; stearic acid; behenic acid; linoleic acid; oleic acid; linolenic acid; 1-terpineol; (-)-α-pinene; linalool; 4-terpineol; α-terpineol; (E)-2-decenal; α-damascenone; α-elemene; trans-caryophyllene; 2,5-di-tert-amylquinone; α-humulene; nerolidol; cis-3-hexenyl benzoate; (-)-caryophyllene oxide; cis-ocimene; borneol; α-eudesmol; octadecane; humulene oxide; camphor; heptadecane; phytone; nonadecane; hexadecanoic acid; eicosane; heneicosane; docosane; tricosane; eicosyl–oleic acid ester; para-cymene; germacrene A; α-gurjunene; α-guaiene; γ-selinene; α-selinene; (-)-α-panasinsen; palustrol; globulol; viridiflorol; tetracosane; pentacosane; [R-[R,R-(E)]]-3,7,11,15-tetramethyl-2-hexadecen-1-ol; 3-methyl-6-(1-methyleth yl)-2-cyclohexen-1-one; elemol; α-copaene; isocaryophyllen; α-cadinene; δ-cadinol; cis-guriune; guaiol; phytol isomer; octadec-9-enoic acid octyl ester; octacosane; δ-selinene; pivalic acid octyl ester; α-terpinolene; germacrene B; 1,2,2,6,8-pentamethyl-7-oxabicyclo[4.3.1]dec-8-en-10-one; heptacosane; nonacosane; octadecanoic acid ethyl ester; 1,8-menthadien-4-ol; γ-cadinene; germacrene D; eremophilene; α-cadinol; trans-cadinol; cuminol; hexadecane	[18]
Mango leaves	Aqueous extract	Acarbose; manindicin A; manindicins B; mangiferin; norathyriol	[31]
Mango leaves	Aqueous extract	Acetaldehyde; 2-hydroxyacetophenone; 2-furanmethanol; furfural; phenol; 2,3-Dihydro-3,5-dihydroxy-6-methyl-4H-pyran-4-one; oleic acid; o-catechol; hydroquinone; pyrogallol	[32]
Mango leaves	Ethanol extract, dichloromethanic fraction	Apigenin; ferulic acid; quercetin; gallic acid; caffeic acid	[33]
Mango leaves	-	Iriflophenone-3-C-β-glucoside; mangiferoside A; foliamangiferoside B; mangiferoside A1; foliamangiferoside A4; mangiferoside A2; foliamangiferoside A3; maclurin-3-C-β-D-glucoside; 2,4,4′,6-tetrahydroxy-3′-methoxy-benzophenone-3-C-β-D-glucopyranoside; maclurin 3-C-(2-O-galloyl)-β-D-glucoside; maclurin 3-C-(6″-O-p-hydroxybenzoyl)-β-D-glucoside; maclurin 3-C-(2,3-di-O-galloyl)-β-D-glucoside; maclurin 3-C-(2″-O-p-hydroxybenzoyl-6″-O-galloyl)-β-D-glucoside; maclurin 3-C-(2″-O-galloyl-6″-O-p-hydroxybenzoyl)-β-D-glucoside; maclurin 3-C-(2″,3″,6″-tri-O-galloyl)-β-D-glucoside; iriflophenone-3-C-(2-O-p-hydroxybenzoyl)-β-D-glucopyranoside; iriflophenone 3-C-(2-O-galloyl)-β-D-glucoside; mangiferoside C1; mangiferoside C3; foliamangiferoside C7; foliamangiferoside C6; foliamangiferoside C5; foliamangiferoside C2; foliamangiferoside C4; iriflophenone 3-C-(2″,6″-di-O-galloyl)-β-D-glucoside; iriflophenone 3-C-(2″,3″,6″-tri-O-galloyl)-β-D-glucoside; iriflophene; 2,4′,6-trihydroxy-4-methoxybenzophenone; iriflophenone-2-O-β-D-glucopyranoside; 2,4′,6-trihydroxy-4-methoxybenzophenone-2-O-β-D-glucopyranoside; 4,4′,6-trihydroxybenzophenone-2-O-α-L-arabinofuranoside; 4,4′,6-trihydroxybenzophenone-2-O-(2″),3-C-(1″)-1″-desoxy-β-fructopyranoside; 4′,6-dihydroxy-4-methoxybenzophenone-2-O-(2″),3-C-(1″)-1″-desoxy-β-fructopyranoside; 4,4′,6-trihydroxybenzophenone-2-O-(2″),3-C-(1″)-1″-desoxy-β-fructo-furanoside; aquilarinoside A; 4′,6-dihydroxy-4-methoxybenzophenone-2-O-(2″),3-C-(1″)-1″-desoxy-α-L-fructofuranoside	[26]

**Table 4:** Health promoting activities of mango leaves.

Variety of Mango	Type of Extract	Bioactive Compounds Identified	Type of Cell Lines/Type of Study	Major Findings and Molecular Mechanisms of Action	References
Anti-cancer activities					
Kent	Extract prepared by pressurized liquid extraction and enhanced solvent extraction	Homo-mangiferin, methyl gallate, gallotannins	MDA-MB-231, MCF7, MCF10	Leaf extracts with high concentration of homomangiferin and methyl gallate were found more effective against MDA-MB-231 cells, while gallotannins showed cytotoxicity against MCF7 cells at IC_50_ > 200 µg/ml	[41]
Okrong	Ethanol extract	Mangiferin	Lung fibroblast (ATCC CLS 300421,WI-38 VA-13 subline 2RA), skin fibroblast (ATCC CRL1947, CCD-986SK), colon adenocarcinoma (ATCC CCL227, SW 620), gastric carcinoma (ATCC HTB103, Kato-III), liver hepatoblastoma (ATCC HB8065, Hep-G2), bronchogenic carcinoma (ATCC HTB-168TB, Chago K-1), and ductal carcinoma (ATCC HTB20, BT474)	Leaf extracts showed potent cytotoxic activities at IC_50_ >200 µg/mL against all the cell lines	[42]
Anti-diabetic activities					
Young leaves of **Mangifera indica** cv. Anwar Ratol were obtained from a private mango farm, Multan, Pakistan.	Hydro-alcoholic	Mangiferin, Phenolics, and flavonoids	In-vivo (Swiss albino mice with alloxan monohydrate (150 mg/kg *i.p.*) induced diabetes)	Administration of ML extract (550, 750, 950 mg/kg) significantly reduced the postprandial BGL, improved the lipid profile, body weight, and glucose tolerance, and also prevented the β-cells damage.	[49]
Leaves collected in Yuanjiang county, Yunnan province, China.	Distilled Water extract further subjected to chromatography over various columns	Two new benzophenone (Manindicins A & B) and two xanthones (Mangiferin & Norathyriol)	In-vitro	Norathyriol exhibited strong inhibition of α-glucosidase activity with an IC_50_ of 4.22 ± 0.19µg/mL, which was 4-fold lower than the commercial acarbose (16.28 ± 1.22 µg/mL).	[31]
Leaves collected from Guangdong Pharmaceutical University, China	70% ethanol-water extract	Five benzophenones and seventeen flavonoids	In-vitro	Among all isolated compounds, quercetin-3-O-α-L-rhamnoside (IC_50_ 76.69 ± 34.79 µg/mL) and quercetin (IC_50_ 31.17 ± 5.06 µg/mL) displayed a stronger inhibition of α-glucosidase than acarbose (IC_50_ 119.59 ± 6.00 µg/mL).	[30]
Leaves collected from Guangdong Pharmaceutical University, China	70% ethanol-water extract	Benzophenone glycosides	In-vitro	Novel 2,4,4′,6-tetrahydroxy-3′-methoxybenzophenone-3-C-β-D-glucopyranoside (IC_50_ 97.44 ± 20.29 µg/mL), arjunolic acid (IC_50_ 117.09 ± 25.00 µg/mL), and actinidic acid (IC_50_ 144.72 ± 8.12 µg/mL) displayed the potent α-glucosidase inhibitory	[87]
Leaves of **Mangifera indica** cv. Okrong collected in Thailand	Ethanol extract	Mango leaf extract and Mangiferin	In-vitro	MLE exhibited the inhibition of yeast α-glucosidase (IC_50_ 50.3 µg/mL) > rat α-glucosidase (IC_50_ 1452.8 µg/mL) > pancreatic α-amylase (IC_50_ 2284 µg/mL). Mangiferin exhibited the inhibition of yeast α-glucosidase (IC_50_ 581.3 µg/mL) > rat α-glucosidase (IC_50_ 433.3 µg/mL) > pancreatic α-amylase (IC_50_ 1048.5 µg/mL).	[42]
Tender and mature leaves of **Mangifera indica** cv. Totapuri collected from GKVK, Bangaluru.	70% Methanol	Tender and mature leaf extract (TLE and MLE)	In-vitro and in-vivo (Wister Albino rats)	Administration of TLE and MLE (500mg/kg body weight) showed potent inhibition α-amylase (IC_50_ 22.01 µg/mL) and α-glucosidase (IC_50_ 21.03 µg/mL) respectively.	[52]
Antioxidant activity					
Mango leaves extract	Supercritical process (CO2/methanol (50%) at 120 bar and 100 °C)	Polyphenols (iriflophenone, mangiferin, and gallic acid)	In vitro	Potent antioxidant (AAI = 3.28 ± 0.1 µg DPPH/µg extract).	[88]
Mango leaves extract	Water	Polyphenols (mangiferin)	In vivo	Stimulated concentrations of Catalase activity (CAT) (32.4 ± 1.9 U CAT mg Ptn−1) and (Total antioxidant capacity) TAC (0.27 ± 0.01 mM Trolox), nearly doubling the obese group (OB) and (non-obese group) CG values	[74]
Mango leaves extract	Water	Polyphenols	In vitro	IC_90_ values for DPPH and FRAP assay were 156.08 and 5.44 μg/mL, respectively, at 500 μg/mL concentration of extract	[48]
Antimicrobial activity					
Mango leaves extract	Aqueous extract And Chloroform extract	Alkaloids, tannins, terpenoid, anthraquinones, reducing sugar, amino acid, flavonoids, steroid, saponins, cardiac glycosides, resin, phenols.	Manifestation of antimicrobial activity of aqueous and chloroform extracts against Methicillin Resistant *Staphylococcus aureus*	The chloroform extract with high range of zone of inhibition (14–17 mm) manifested to have higher antibacterial property against the bacteria with respect to to aqueous extract.	[89]
Mango leaves extract	Ethanolic extract (50% and 100%) Hydroalcoholic extract (50% and 100%)	Polyphenols tannins, terpenoids	Estimation of antimicrobial activity of ethanolic and hydroalcoholic extracts against *Staphylococcus aureus* ATCC 6538	50% and 100% ethanol extract—small zone of inhibition—21.4−24.3 ± 0.8 mm Hydroalcoholic extract (50% and 100%) with larger zone of inhibition 24.7 − 33.4 ± 1.2 mm, therefore higher antimicrobial activity than ethanol extract	[90]
Leaf Extract	Ethanolic extract	Alkaloids, anthranol, glycosides, saponins, triterpenes, phenol, flavonoids	Manifestation of antimicrobial activity of ethanolic extract against *Shigella flexneri*, *Pseudomonas fluorescens*, *Escherichia coli*, *Staphylococcus* *Aureus*, and *Bacillus* spp.	Ethanol extract showed no inhibitory effect on *Staphylococcus aureus* and not so strong inhibitory effects against the other four organisms. The MIC ranges from 12.4 to 26 mg/mL with zones of inhibition ranges from 18 to 25 mm	[91]
Leaf Extract	Ethanolic extract	Polyphenols tannins, terpenoids	Estimation of antimicrobial activities of ethanolic leaf extracts of mango and its use in bio control of food spoilage microorganisms	Ethanolic extracts of mango leaves had the best MIC against *E. coli* (6.25 mg/mL), *P. aeruginosa* (12.5 mg/mL) and *S. aureus*, *L. casei* and *Listeria monocytogenes* (25 mg/mL)	[92]
Hepatoprotective and anti-obesity activities					
Young leaves of var. Ubá from Zona da Mata area, Brazil	water	Mangiferin	In-vivo (male Wistar rats, weight = 200 ± 50 g, age = 60 days, fed a high-fat diet)	Application of MLT (25 mL/day for 8 weeks) supressed the increase in weight, maintained lower levels oftriacylglycerols, alanine aminotransferase, and total cholesterol. Alters the gene expression, i.e., reduced expression of NF-κB p65 and activated PPARα expression, which exhibited hepatoprotective activity	[74]
Fresh leaves of mango cultivars were collected from Krishnagiri, India	Methanolic	3β-taraxerol	In-vitro pancreatic cholesterol esterase inhibition assay for bioactivity guided fractionation (BAGF)	3β-taraxerol (IC_50_ value = 0.86 µg ml^−1^) exhibited hypocholesterol activity	[75]
Fresh leaves of *Mangifera indica* L. var. Sindoora were obtained from Krishnagiri, India	Methanolic	3β-taraxerol, mangiferin, and iriflophenone-3-C-β-glucoside	In-vivo Male albino Wistar rats	Application of MLE from 21th day to 42th day (90 mg/kg) under six weeks of study significantly reduces plasma triglycerides.	[77]
Fresh leaf samples of *Mangifera indica* L. var Ataulfo were obtained from San Blas, Nayarit, Mexico	Methanolic	Mangiferin	In-vivo Male Wistar rats (8-week-old)	Application of MLE (200 mg/kg) significantly reduces level of cholesterol and triglycerides and enhanced HDL level	[78]
